# A Case of Dysphagia Due to Large Osteophytic Lesions in the Cervical Spine: A Conservative Approach

**DOI:** 10.7759/cureus.59011

**Published:** 2024-04-25

**Authors:** Christos Lyrtzis, Alexandros Poutoglidis, Athina Stamati, Nikolaos Lazaridis, George Paraskevas

**Affiliations:** 1 Department of Anatomy and Surgical Anatomy, Aristotle University of Thessaloniki, Thessaloniki, GRC; 2 Department of Otorhinolaryngology-Head and Neck Surgery, “G. Papanikolaou” General Hospital, Thessaloniki, GRC; 3 Department of Anatomy and Surgical Anatomy, School of Medicine, Aristotle University of Thessaloniki, Thessaloniki, GRC; 4 Department of Anatomy and Surgical Anatomy, Faculty of Health Sciences, Aristotle University of Thessaloniki, Thessaloniki, GRC

**Keywords:** conservative treatment, larynx, head and neck, osteophytes, dysphagia

## Abstract

Dysphagia is a common symptom with various underlying etiologies, making its management challenging even for experienced physicians. The presence of osteophytes in the cervical spine may often impede swallowing, displace the larynx, and cause a sore throat. We describe a case of an 85-year-old male who presented with a two-year history of progressive dysphagia, exacerbated over the last two months, especially with solid foods and liquids, prompting an ENT evaluation. Despite prior investigations, including normal gastroscopy and empirical pain management, further assessment revealed bulging masses in the hypopharynx indicative of cervical osteophytes. Conservative management, including speech and swallow therapy, dietary modifications, and pharmacological interventions, resulted in significant symptom improvement without surgical intervention. This case demonstrates the effectiveness of conservative treatment measures in treating dysphagia caused by cervical osteophytes, emphasizing the significance of a multidisciplinary approach for optimal patient care.

## Introduction

Dysphagia, or difficulty swallowing, is a multifactorial condition with various underlying etiologies, making its management challenging even for experienced physicians [1]. Dysphagia can arise from various causes, including structural abnormalities such as tumors or strictures in the esophagus, neurological conditions affecting the swallowing mechanism, or muscular disorders impairing swallowing function [2]. Additionally, inflammation, trauma, or degenerative changes in the cervical spine can also lead to dysphagia by compressing or obstructing the esophagus [2]. Cervical osteophytic lesions present a unique and relatively uncommon cause. Osteophytes, also known as bone spurs, are bony outgrowths that commonly develop at the margins of vertebral bodies as a result of degenerative changes in the cervical spine [3]. While often asymptomatic, in some cases, these osteophytes can exert pressure on adjacent structures, including the esophagus, leading to dysphagia. Aggressive surgical treatment with osteophytectomy is controversial and should be considered the last resort [3]. We present a case of an elderly patient with dysphagia due to major osteophytes of the cervical spine that were treated successfully with conservative measurements.

## Case presentation

An 85-year-old male presented to our Ear, Nose, and Throat (ENT) Department with the chief complaint of difficulty swallowing and neck stiffness. Dysphagia had been initiated two years ago but worsened over the last two months, especially during meals that included solid foods with occasional aspirations and choking on liquids. Despite a previous normal gastroscopy and prescription of painkillers, symptoms persisted, prompting further evaluation. After an orthopedic consultation, he was referred for an ENT evaluation. According to his medical history, the patient had diabetes mellitus and increased blood pressure.

**Figure 1:**
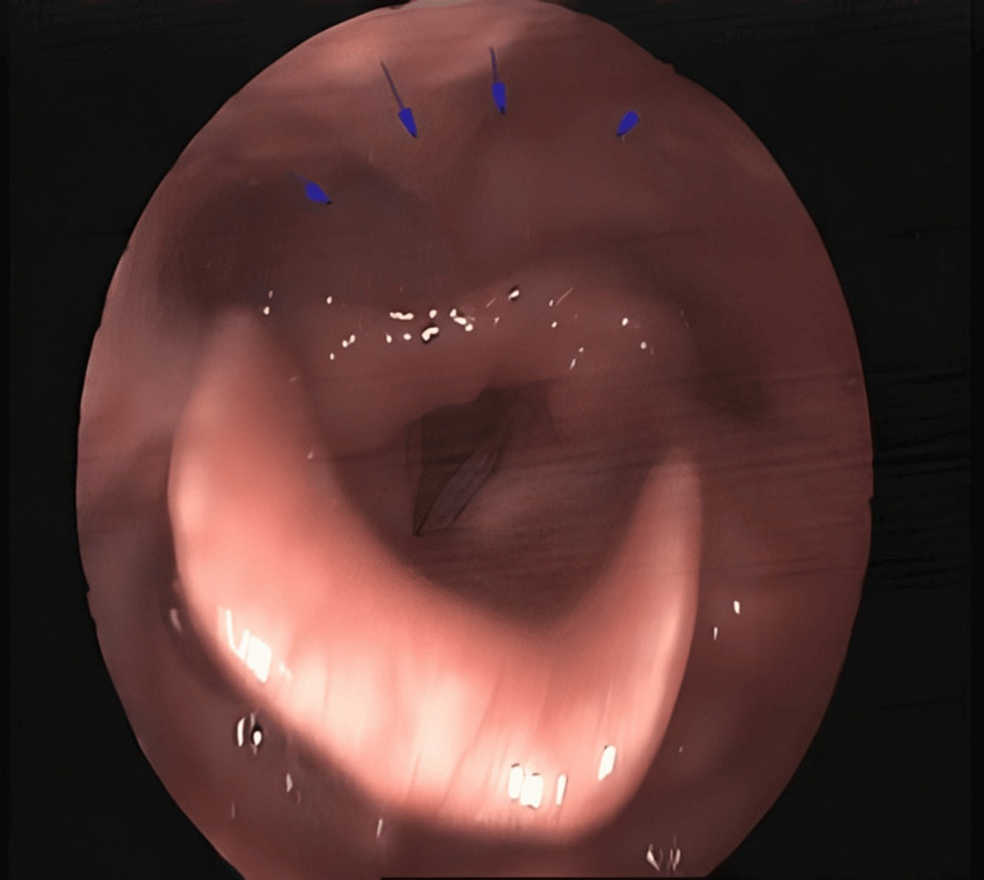
Anterior displacement of the right hemilarynx in flexible endoscopy. Arrows point to the area of displacement

**Figure 2:**
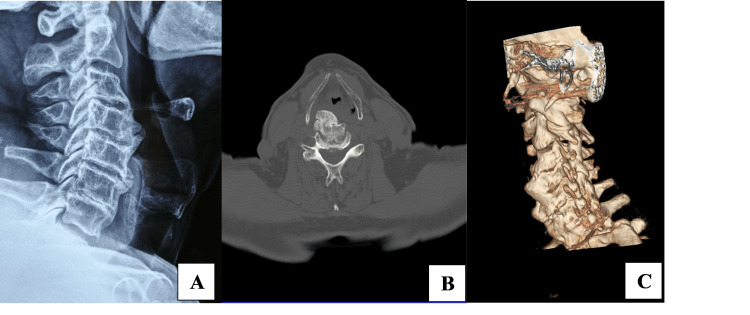
The presence of cervical spine osteophytes in a) cervical spine X-ray, b) 3D reconstruction computed tomography, and c) computed tomography

Flexible endoscopy revealed the presence of a bulging mass in the posterior wall of the hypopharynx with anterior displacement of the posterior right part of the larynx (Figure 1). Fiberoptic endoscopic evaluation of swallowing (FEES) was indicative of poor swallowing and aspiration in liquids. Subsequent radiologic testing with X-rays and computed tomography (CT) with 3D reconstruction identified two large osteophytic lesions in the bodies of the fourth and fifth cervical vertebrae (Figure 2). Therefore, the diagnosis of dysphagia due to anterior osteophytes of the cervical spine was confirmed.

The symptoms were treated conservatively due to the age of the patient and the risk of complications after surgery. The patient was referred for speech and swallow therapy to manage his meals and avoid aspiration and choking. The speech therapist suggested dietary restrictions and modified meals. In addition, antireflux medication and muscle relaxants were prescribed for better symptom control for two months. After two months, the patient reported significant relief of symptoms. A new FEES indicates better management of liquids with minimal aspirations. The patient was encouraged to continue his speech and swallow therapies. A monthly follow-up for a year was also suggested for better surveillance of his dysphagia. In adherence to ethical standards, the patient in this case report provided informed consent, demonstrating their complete comprehension and voluntary willingness to disclose their medical details.

## Discussion

Dysphagia is a complicated medical condition with a wide range of probable causes, including anatomical abnormalities and neurological impairments [2]. In this case, dysphagia was caused by extensive osteophytic lesions in the cervical spine, which is a rarer etiology compared to esophageal strictures or neurological diseases. Specifically, this case study presents an 85-year-old male with a two-year history of progressive dysphagia, exacerbated over the last two months, particularly with solid foods and liquids, prompting an ENT evaluation. Despite a previous normal gastroscopy and empirical pain management, further investigation revealed bulging masses in the hypopharynx indicative of cervical osteophytes. Management of dysphagia secondary to cervical osteophytes posed unique challenges, particularly in an elderly patient with comorbidities such as diabetes mellitus and hypertension. Given the patient's advanced age and the associated risks of surgical intervention, a conservative treatment strategy was adopted. Importantly, it's worth noting that the patient's condition was managed without resorting to surgical intervention. This included speech and swallow therapy to enhance swallowing mechanics and prevent aspiration, dietary modifications to optimize oral intake, and pharmacological measures such as antireflux medication and muscle relaxants to alleviate symptoms.

The literature on dysphagia caused by cervical osteophytes is limited, suggesting its rarity in comparison to other causes of dysphagia. Existing research and especially case reports, however, emphasize the need to include cervical spine pathology in the differential diagnosis of dysphagia, particularly in elderly people with degenerative abnormalities in the spine [4,5]. The results of Seidler et al. demonstrate that dysphagia secondary to ventral osteophytes of the cervical spine presents with varied clinical and radiographic manifestations, with osteophyte location and size playing significant roles in determining the severity of symptoms and influencing the need for surgical intervention [6]. According to a systematic review, osteophytes primarily affect old males and include the vertebrae C3-C5 [7]. Denko C. and Malemud C. demonstrated the role of insulin in bone formation as an anabolic agent [8]. Therefore, diabetes mellitus should also be considered a risk factor.

Management of dysphagia with surgical resection of osteophytes seems to be more effective than conservative treatment for symptom relief [9]. However, this modality poses a significant risk of complications, including Horner syndrome, pharyngocutaneous fistula, infection, and vocal cord palsy [10]. In addition, osteophyte recurrence with subsequent dysphagia has also been reported by Miyamoto et al. in two out of seven patients [11]. Although Park et al. demonstrate that surgical treatment of anterior osteophytes causing dysphagia can lead to overall improved functional swallow outcomes, a high preoperative Functional Outcome Swallowing Scale (FOSS) score may indicate a less favorable postoperative outcome [12]. According to Choi et al., in patients with dysphagia caused by anterior cervical osteophytes (ACOs), the thickness of the osteophytes was significantly associated with the choice of surgical treatment, while dysphagia severity at the pharyngeal phase and osteophyte thickness were important considerations in determining treatment options [13]. Finally, Egerter AC et al. suggested the application of bone wax to resected osteophytes to avoid future recurrence [9]. Therefore, a judicious approach to treatment selection is paramount, considering both the potential benefits and risks associated with invasive interventions.

In addition to the presented case of dysphagia attributed to large osteophytic lesions, it is essential to acknowledge other conditions such as diffuse idiopathic skeletal hyperostosis (DISH) and ankylosing spondylitis, which can also manifest with similar symptoms related to bony tissue proliferation in the cervical spine [14,15]. Treatment for dysphagia associated with conditions like DISH and ankylosing spondylitis involves a multidisciplinary approach. Conservative measures such as physical therapy, NSAIDs, and lifestyle modifications are often employed initially to alleviate symptoms and improve swallowing function. Surgical intervention may be considered for refractory cases, with treatment strategies tailored to the underlying pathology and individual patient needs [14,15].

Our experience indicates that surgery should be the last resort, especially for elderly people with comorbidities. Conservative treatment offers significant pain relief and should always be the first step in managing dysphagia.

## Conclusions

In conclusion, dysphagia secondary to cervical osteophytes represents a unique clinical entity necessitating a multidisciplinary approach for accurate diagnosis and tailored management. Further research is warranted to elucidate optimal treatment algorithms and long-term outcomes in this patient population, thereby enhancing clinical care and improving patient quality of life. This case report highlights the efficacy of non-invasive approaches in treating dysphagia associated with cervical osteophytes. Regular follow-up examinations allowed for more accurate monitoring of symptom progression and treatment response, ensuring ongoing optimization of therapeutic interventions.
